# Supplementary material for: Acquisition of handwriting in children with and without dysgraphia: A computational approach
Source: PLoS One. 2020 Sep 11;15(9):e0237575. doi: 10.1371/journal.pone.0237575 (PMC7485885; doi:10.1371/journal.pone.0237575)
Supplement: Attachment — Submitted filename: Response to Reviewers_ PONE-D-19-34910 DC.docx [file pone.0237575.s001.docx]

PONE-D-19-34910

Acquisition of handwriting in children with and without dysgraphia: a computational approach

RESPONSE TO REVIEWERS

RESPONSE TO REVIEWER #1:

**We thank reviewer 1 for his/her interest and valuable feedbacks regarding our MS. We believe these improved both quality and readability of the revised MS. We addressed all points raised by reviewer 1 as follow.**

**Point 1.** Methods: I did not find any statistical analysis about the agreement.

**We thank reviewer 1 for raising this point that was unclear. As scoring was a continuous variable, we used an Intra class Correlation. We clarified this in the revised MS. It is now pages 4-5 of the revised MS: “*Two junior psychomotor therapists were trained by the same senior psychomotor therapist to score BHK. Then, the 2 juniors therapists annotated independently all BHK both for quality and speed scores. For the 30 least consistent scores (BHK score > 5), the senior therapist scored the BHK. These professionals were blinded to the demographics and clinical characteristics of the children. Scoring included two dimensions: (1) handwriting velocity assessed through the number of characters written in five minutes and (2) handwriting quality on the five first sentences of the text according to 13 items using a semiquantitative method (BHK quality scores: Table 2). We calculated the final inter rater-reliability using intra-class correlation, ICC = 0.97 (95% CI: 0.96-0.98) Finally, according to the normal scores by age measured during the previous validation of the scale [17], we computed a qualitative score (quality of the writing) and a quantitative score (speed of the writing).*”**

**Point 2.** Results: Delete "only" from page 7, line 209 and page 13 line 377.

**This was done accordingly in the revised MS**

**Point 3.** Likewise, delete "As expected" from page 7, line 218 since it was not an objective of the study.

**This was done accordingly in the revised MS**

**Point 4.** Discussion: Delete one of the two "Finally" from page 14 lines 406 and 445.

**These were done accordingly in the revised MS**

RESPONSE TO REVIEWER #2:

**We thank reviewer 2 for his/her interest and valuable feedbacks regarding our MS. We also thank reviewer 2 for his/her careful editing of our MS. We believe these improved both quality and readability of the revised MS. We addressed all points raised by reviewer 1 as follow.**

Overall Comments

**Point 1.** Quantitative vs qualitative dysgraphia – This paper's usage of "quantitative" and "qualitative" is non-standard, so it should be clarified upfront.

**We thank reviewer 2 for this important comment. Indeed, the term qualitative can be confusing. Reviewer 2 is right: all the analyses we performed were quantitative. To clarify that, we replaced when necessary qualitative by quality score which is used for BHK to quantify the quality of writing (as opposed to speed). It is a continuous numerical score described Table 2. We also replaced quantitative by speed.**

**Point 2.** Groups – Why were the comparisons/interactions analyzed with TD vs TD+D groups rather than simply TD vs D? In general, this seems like a very indirect way of analyzing the groups.

**We thank reviewer 2 for this important comment and we understand his/her concern. A said in the introduction, we selected the 12 digital features through machine learning classifying BHK scores as threshold (binary classification). As machine learning is based on statistical occurrences, we already know that the differences between TD vs. D are significant. To clarify our choice in the revised MS, we made the following revisions:**

**1. In the introduction after summarizing the first study we performed to select the digital features through machine learning, we added the following paragraph (page 3 of the revised MS): “*Despite the inherent progressive learning of writing, so far no study using digital features took into account age and had a developmental approach. We still do not know how the selected features classifying children with dysgraphia evolved in TD children. In addition, we don’t know whether their ability to detect children with dysgraphia changed with age.*”**

**2. We also better distinguish the objectives of the study. It is now page 3 of the revised MS: “*In the current study, we aimed to extend our work [24] addressing the effect of age, and the heterogeneity of dysgraphia. Our objectives were the following: 1. First, we aimed to present the learning and acquisition of handwriting from a developmental approach (according to child age). We explored TD children in order to better understand typical development (TD dataset only) and children with dysgraphia (D dataset). 2. Second, we aimed to identify the best features, to diagnose children with dysgraphia (according to age) both using the clinical gold standard method as well as relevant digital features [24]. 3. Third, […]”***

**3. In the method section we explained this choice in terms of statistics. It is now pages 6-7 of the revised MS: “*Since we selected the 12 digital features through machine learning classifying BHK scores as threshold (binary classification), we considered inappropriate to use direct group comparisons (TD vs. D). To take into account the effect of age, and possible interactions between a given digital feature and age, we applied linear regressions considering each feature as a continuous variable to explain the BHK as a continuous variable without consideration of the diagnosis threshold. To do so, each of the 12 digital features was normalized in order to assess their effect in linear regression models.***

***To understand how a given digital feature is explaining or not BHK quality taking into account grade and gender, a linear regression model per feature was created to predict the continuous BHK quality score. This model was adjusted for grade and gender. In the same way, a model was created to predict the BHK speed score. The formulas can be described as follows:***

***To understand how a given digital feature explaining continuous BHK changes according to a child’s grade, a similar model with interaction [grade*Normalized(feature)] was also created. In other words, the model can show the relative importance of a given digital feature to diagnose dysgraphia according to age. As recommended in the BHK manual, we selected the grade rather than the age to assess the effect on education, since the writing process is learned at school and not spontaneously. The formulas can be described as* follows:**

***Since the distribution of the residuals was not normally distributed, a bootstrap analysis (with 10,000 replications) was performed to assess the 95% confidence intervals (95%CI) and p values. These were respectively obtained by BCA (bias-corrected and accelerated) bootstrap and percentile bootstrap with the R boot package. As said previously, we performed these analyses on the TD dataset only to explore how digital features predict writing (BHK) quality and speed in TD children, then on the TD + D dataset to explore how digital features predict writing (BHK) quality and speed in a mixed population that resembles a more realistic situation in the context of school detection of D children.”***

**Point 3.** Figure numbering – Most (all?) figure references need to be fixed. The Results and Discussion were difficult to follow because of the mixed-up numbering.

**We thank reviewer 2 for this. We are sorry for the confusion. All numbering were checked and fixed accordingly**

Line-by-Line Comments

**Point 1.** Abstract: "but present a qualitative different development in terms of static, kinematic, pressure, and tilt feature" – These are quantitative features, so the "qualitative" reference is confusing.

**As requested, we replaced qualitative by quality score. It is a continuous numerical BHK score described Table 2. Also, we replaced quantitative by speed.**

**Point 2.** L15: "Despite correct training" – Please expand on what is considered "correct" training (or reword). I am not sure about [17] as I cannot find an English version, but [19] used 3 months of physiotherapy. Is that considered objectively correct?

**Sorry for misusing correct training. We changed in the revised MS for “*Despite normal education exposure”***

**Point 3.** L66: "with a sensibility of 96.6%" – Is this supposed to be sensitivity?

**As requested, sensibility was changed to sensitivity**

**Point 4.** L106: "Pressure data were carefully calibrated between the two tablets" – Please expand on this calibration. Was this a simple linear mapping, or did it require something more complex? Also, consider moving the description of the pressure comparisons (from the Limitations section) to here

**As suggested, a more precise description of the procedure used for the calibration was added to the manuscript. It is now page 4 of the revised MS: “***The weights X were carefully chosen (from 0g (pen only) to 400g) in order to explore all range of tablet outputs until saturation. The relation between the weight in input (X) and the value returned by the tablet (Y) could be extracted and was found to be very similar for the two tablets (Spearman correlation > 0.99, p < 0.001, mean square error = 0.6). A 4th degree polynomial fit was created to model the function describing the X/Y relation of the first tablet and used on the second to correct the output. After this correction, the spearman correlation was found to be 0.99998 (p << 0.001) and the mean squared error was 5.1x10-3.”*

**Point 5.** L108: "inter-cotator value of 97%" – Is this referring to inter-rater?

**We thank reviewer 2 for this comment. Indeed, we calculated the final inter rater-reliability using intra-class correlation (ICC = 0.97 (95% CI: 0.96-0.98)**

**Point 6.** L211: "We considered the children with qualitative dysgraphia as children with dysgraphia in the D dataset the children having qualitative dysgraphia" – Unclear, please clarify

**We thank reviewer 2 for this comment. After clinical assessment of all BHK tests from our dataset (280 children), we confirmed dysgraphia in all children recruited in special clinics and detected 13 (5.63%) children with dysgraphia among those recruited from regular schools. Speed dysgraphia (slow writing) was observed in 12 children with all of them showing also qualitative dysgraphia (poor quality, legibility). *Thus, we defined the diagnostic category of dysgraphia based on the BHK quality score only.***

**This last sentence was added in the revised version of the MS to make clear our point.**

**Point 7.** L242: "As expected, since all these features already classified in a proper manner dysgraphia on a binary classification in Asselborn et al. [24]" – Unclear, please clarify

**We thank reviewer 2 for this comment. This point is related to what we said and clarified previously (see point 2 response to overall comments). To clarify this point we changed the sentence in the revised MS as follow (page 5): “*In this work, we only used the features that were found to be the most important in the aforementioned random forest model according to the Gini importance metric [24]. This means that all the features were significantly different between TD and D based on a binary diagnostic classification (BHK threshold). As expected, all digital features were significantly associated with continuous BHK quality score when the models were applied on TD children and children with dysgraphia.*”**

\# Copyedits

**Point 1.** L9: "handwriting initially evolves first on a qualitative level" – Remove either "first" or "initially" (redundant).

**This was done accordingly in the revised MS**

**Point 2.** L34/L408/L417: "Deuel et al. [23]" – These should be Deuel [23] (without et al).

**This was done accordingly in the revised MS**

**Point 3.** L62: "typically developing (TD) and children with dysgraphia" – Reword to "typically developing (TD) children and children with dysgraphia" or "children with typical development (TD) and with dysgraphia."

**This was done accordingly in the revised MS**

**Point 4.** L128/L142/L156/L166: "They regroup features" – Using "they" is a bit awkward here. Consider "these" or something similar.

**This was done accordingly in the revised MS**

**Point 5.** L159: "Mean Speed Of Pressure Change" –"Of" should be lowercase.

**This was done accordingly in the revised MS**

**Point 6.** L192: "the distribution of the residuals were not normally distributed" – "Were" should be "was."

**This was done accordingly in the revised MS**

**Point 7.** L199: "theoretical classification of Deuel" – In-text citation [23] should be included.

**This was done accordingly in the revised MS**

**Point 8.** L210: "and all of them showing" – Reword to "with all of them showing" or "and all of them showed."

**This was done accordingly in the revised MS**

**Point 9.** L219: Extra space before the final period.

**This was done accordingly in the revised MS**

**Point 10.** Table 4: Extra parenthesis after "quality" and missing parenthesis after "dataset."

**This was done accordingly in the revised MS**
