# Supplementary material for: Acquisition of handwriting in children with and without dysgraphia: A computational approach
Source: PLoS One. 2020 Sep 11;15(9):e0237575. doi: 10.1371/journal.pone.0237575 (PMC7485885; doi:10.1371/journal.pone.0237575)
Supplement: Attachment — Submitted filename: Response to Reviewers.docx [file pone.0237575.s002.docx]

PONE-D-19-34910

Acquisition of handwriting in children with and without dysgraphia: a computational approach

RESPONSE TO REVIEWER #1:

We thank reviewer 1 for his/her interest and valuable feedbacks regarding our MS. We believe that they improved both quality and readability of the revised MS. We addressed all points raised by reviewer 1 as follow.

Reviewer #1:

**Point 1. Page 2: line 15: In the sentence, “Despite normal education exposure, 5% to 10% of children never reach a sufficient 1 level of automation in handwriting” delete "normal".**

As requested we changed the sentence accordingly.

**Point 2.** **Page 3: line 61: delete "have"**

As requested we deleted the word ‘have’ in the sentence accordingly.

**Point 3.** **I have problems to understand the number of participants.**

We have detailed the number of participants and group allocation in the Material and Method section and in a diagram flow shown in Figure 2. However, the reviewer is perfectly right there was a mistake in the transcription of numbers when we referred to 298 children. In fact pages 3 and 4 we corrected to the right total number N=280. We thank you for pointing this error.

**Point 4.** **give actual " p values"**

Given the complexity of the MS we chose to be conservative with the p values to ease reading (asproposed by PlosOne manuscript recommendation). All p values are reported, but they are shown in all tables 3, 4, 5, 6, 7 reporting the multivariate models and table 8 reporting the cluster analysis.

Table 1 is a description of the participants and table 2 lists the items used in the BHK test and the automatic features that we extracted. There is no comparison, so no p values need to be reported.

**Point 5. CHECK the results in the text: In results section (2.1) the first aim does not fit with what was mentioned in page 3. Please reorganize sentences and aims. Likewise, results for aim 1 were mentioned but not for the rest of aims.**

We agree with the reviewer and to help following our aim in the results section, we repeated them accordingly. We added in the revised version of the MS at the beginning of section 2.2 the following sentence: “*Our second aim was to identify the best features to diagnose children with dysgraphia both using BHK scores (the clinical gold standard method) and relevant digital features, and to explore how the relevant digital features had statistical interaction with age*.”

In addition, we added at the beginning of section 2.3.: “*Our third aim was to explore whether we could define a new classification of dysgraphia based on our automated features. Hence, most of the classifications...*”

RESPONSE TO REVIEWER #3:

We thank reviewer 3 for his/her interest and valuable feedbacks regarding our MS. We believe that they improved both quality and readability of the revised MS. We addressed all points raised by reviewer 3 as follow.

Overall Comments

**Point 1.** **The discussion about anxiety with dysgraphia needs to explain further.**

We thank reviewer 3 for this remark. We added in the introduction two sentences and a reference to develop on the link between dysgraphia and anxiety disorders. It is now page 4 of the revised MS: “*Hence, in children with dysgraphia, the low performance in writing induces negative comparisons with others and self-criticizing. As a consequence, it increases school-performance anxiety and can lead to an increased trait anxiety that can persist until adolescence particularly in boys (Sigurdsson, 2002)*”.

Sigurdsson, E., Van Os, J., & Fombonne, E. (2002). Are impaired childhood motor skills a risk factor for adolescent anxiety? Results from the 1958 UK birth cohort and the National Child Development Study. *American Journal of Psychiatry*, *159*(6), 1044-1046.

**Point 2. The use of pen tablets affect vibration on the user is there a description of it? Does this affect the results of the research?**

We thank reviewer 3 for raising this point that was not clear enough obviously. Our manuscript does not study vibration. Some automatic features are related to tremor of the pen extremity but are not related to clinical tremor or vibration. We believe that we induced a confusion when we explain in the discussion section that individuals who write on a paper sheet and individuals who write on the screen of a tablet may have different writing sensation (often call friction of the pen). We changed the related sentences to make it clear. It is now page 15 of the revised MS: “*Several studies have shown that the writing sensation (often called friction of the pen) of individuals who write with a pen on a sheet of paper might be very different from the one of individuals who write with a stylus on the surface of a digital tablet [33]. To generalize our results with a stylus/tablet setting, we need first to check whether the same set of features remains relevant. A new database of handwriting traces should be acquired directly on digital tablet with a stylus to investigate whether generalization of our results is possible in this setting.*”

**Point 3. The author uses semi-quantitative, quantitative, and qualitative. This three method makes the reader confused about the research procedures with three ways, and what for the three uses? Qualitative scoring in this study is not clarified by what method and for what generalizations?**

We thank again reviewer 3 for raising this point. Hence, we only used quantitative measures (meaning scores) in this study. Otherwise we could not perform linear regressions. The confusion comes from the BHK test that produces 2 scores: one for handwriting speed and the other for handwriting quality. To avoid further confusion, we systematically revised the MS to specify handwriting quality score when needed instead of quality alone as despite a first round of revision we left some occurrences, and we are sorry for that. The changes appear in the ‘revised version modification in yellow’ document.

**Point 4. Pen tablet calibration and the tendency of some students for tablet surface vibrations to find a solution in its generalization can be of equal value when calculated.**

Again, we believe we introduced a confusing argument in the discussion. See previous response to point 2.

**Point 5. This research has a good novelty but has weaknesses in the methods and devices used. The exposure to the use of 3 types of ways was not reinforcing in the revised paper.**

We thank reviewer 3 for this positive remark. Again when one use automated features it is important to have them anchored in clinical practice. When it comes to handwriting as we explained it in the introduction, the BHK test is the clinical gold standard and produces 2 score of equal importance that are the quality and the speed of handwriting. Pupils at school are expected to master both.

**Point 6. Research on the use of different pen tablets in previous studies does not exist, so this study is a new study using two different devices in series; this needs further discussion.**

We thank reviewer 3 for this important remark. We gave in the discussion the main measures and controls we performed to show the minimal impact of using two different models of the same Wacom tablet. For further technical discussion we invite readers to look at the following paper: Asselborn T, Gargot T, Kidzinski Ł, Johal W, Cohen D, Jolly C, et al. Reply: Limitations in the creation of an automatic diagnosis tool for dysgraphia. npj Digital Medicine. 2019;2(1):37.
